# Durability of Transcatheter Heart Valves: Standardized Definitions and Available Data

**DOI:** 10.3390/jcm10184180

**Published:** 2021-09-16

**Authors:** Ines Richter, Holger Thiele, Mohamed Abdel-Wahab

**Affiliations:** Heart Center Leipzig, Department of Cardiology, University of Leipzig, Strümpellstraße 39, 04289 Leipzig, Germany; ines.richter@medizin.uni-leipzig.de (I.R.); holger.thiele@medizin.uni-leipzig.de (H.T.)

**Keywords:** aortic valve stenosis, diagnosis, surgical aortic valve replacement, transcatheter aortic valve replacement, long-term outcome, durability

## Abstract

Transcatheter aortic valve replacement is a well-established alternative to surgical aortic valve replacement in high-risk patients with severe symptomatic aortic stenosis. Currently, this technique is shifting towards younger patient groups with intermediate- and low-risk profile, which raises the question about long-term durability. Despite acceptable results up to 5 years, little is currently known about valve performance beyond 5 years. Since valve deterioration, thrombosis and endocarditis seem to be the main factors affecting valve durability, precise and widely accepted definitions of these parameters were stated by the European Association of Percutaneous Cardiovascular Interventions (EAPCI) in 2017, followed by the Valve in Valve International Data (VIVID) group definitions in 2018 and the Valve Academic Research Consortium 3 (VARC-3) definitions in 2021. Until the introduction of these definitions, interstudy comparisons were difficult due to missing uniformity. Since the release of these recommendations, an increasing number of studies have reported their data on long-term durability using these new criteria. The aim of the present article is to discuss the current definitions on bioprosthetic valve durability, and to summarize the available data on long-term durability of transcatheter aortic valves.

## 1. Introduction

Aortic stenosis (AS) is one of the most common valvular heart diseases in industrialized nations [1]. While transcatheter aortic valve replacement (TAVR) has become the treatment of choice for older patient groups or patients at prohibitive risk for surgical aortic valve replacement (SAVR), treatment options for younger patients or patients with low surgical risk are more variable, and the interplay between life expectancy and durability of prosthetic heart valves becomes a central consideration.

Since two large, randomized trials supported the use of TAVR in patients with intermediate surgical risk [2,3], and the randomized PARTNER 3 as well as the Evolut Low Risk trials showed encouraging results in lower risk patients and younger population groups [4,5], TAVR is expected to further expand into intermediate-risk and low-risk patient cohorts. While currently available studies on transcatheter heart valve (THV) durability showed excellent mid-term results, the key issue for considering TAVR in younger and lower risk patients is limited information on long-term durability.

In 2017 and 2018, the European Association of Percutaneous Cardiovascular Interventions (EAPCI) (endorsed by the European Society of Cardiology (ESC) and the European Association for Cardio-Thoracic Surgery (EACTS)) and the Valve in Valve International Data (VIVID) group introduced standardized definitions on bioprosthetic valve failure (BVF) and structural valve deterioration (SVD). Very recently, the Valve Academic Research Consortium (VARC) released their third set of endpoint definitions, which also included a specific section on bioprosthetic valve dysfunction and failure [6]. Since the release of these standardized definitions, an increasing number of studies reported their data on long-term durability using the new definitions.

The aim of the present article is to review contemporary definitions and to summarize available data on long-term durability of THV using these criteria.

## 2. Definitions of Valve Durability

As described above, the correct reporting of valve durability underlies clear definitions. Historically, bioprosthetic valve durability in terms of SVD was mainly described within the surgical field and was considered as an acquired intrinsic abnormality. It was defined as valve-related death, re-do surgery or valve-in-valve implantation, even though the need for reoperation does not necessarily imply SVD and the presence of SVD does not always lead to reoperation. Specific definitions and criteria on reoperation and/or SVD were not provided. This substantially underestimated the incidence of SVD [7,8]. 

Different definitions and criteria used in the past have rendered interstudy comparisons difficult. In addition, early TAVR patients mainly belong to octogenarian age and the implanted heart valves could outlive their patients, which makes death a well-known competing risk for durability investigations. To address these issues, the EAPCI presented standardized criteria to define BVF, with the aim to generate uniformity in data reporting of future studies [8]. This was the first attempt to provide harmonization within the field of TAVR studies. The group included interventionalists, surgeons and echocardiographers, who agreed on the proposed definitions.

### 2.1. EAPCI Consensus Statement

In 2017, the EAPCI introduced the terms “bioprosthetic valve dysfunction” (BVD) and “BVF”. BVD comprises four modes of dysfunction: SVD, non-structural deterioration (NSVD), thrombosis and endocarditis. BVF can occur in the setting of SVD or as processes unrelated to SVD. It includes (i) BVD on autopsy as a very likely cause of death, or “valve-related death” without confirmatory autopsy, (ii) aortic valve reintervention (valve-in-valve TAVR, paravalvular leakage (PVL) closure or SAVR) and (iii) severe hemodynamic SVD.

SVD, as defined by the EAPCI, can be differentiated into morphological SVD and moderate or severe hemodynamic SVD. Morphological SVD is found mainly by imaging or autopsy and includes transformations in the leaflet integrity (torn or flail causing aortic regurgitation (AR)), the leaflet structure (thickening and/or calcification), the leaflet function (impaired mobility resulting in AS and/or AR) and strut/frame (fracture or failure).

Hemodynamic SVD can be further divided into moderate or severe SVD. Moderate SVD is defined by a mean gradient of ≥ 20 and < 40 mmHg and/or a mean gradient change of ≥10 and <20 mmHg from hospital discharge or within the first 30 days after implantation or a new or worsening (>1+/4+) of intraprosthetic AR. Severe hemodynamic SVD includes a mean gradient of ≥40 mmHg and/or a mean gradient increase of ≥20 mmHg from hospital discharge or within the first 30 days after implantation or a new or worsening (>2+/4+) of intraprosthetic AR. Hemodynamic SVD is diagnosed by means of echocardiography and can exist even without evidence of morphological SVD (isolated hemodynamic dysfunction).

NSVD is caused by extrinsic factors resulting in valve changes. The main factors include moderate/severe patient-prosthesis-mismatch (PPM), moderate/severe PVL, device malpositioning and abnormal frame expansion. Since PPM manifests mainly with elevated gradients, PPM may be difficult to distinguish from SVD, but should be seen separately from SVD. In PPM, the abnormal haemodynamics are present early from the time of implantation. The leaflet morphology is normal, and the gradients and hemodynamics would not change during follow-up [7]. Moderate PPM can be defined as an effective orifice area (EOA) > 0.65 cm^2^/m^2^ and ≤0.85 cm^2^/m^2^. Severe PPM can be defined as ≤0.65 cm^2^/m^2^ [9]. In obese patients (BMI ≥ 30 kg/m^2^), other cut-off values may be used to define moderate PPM (>0.60 cm^2^/m^2^ and ≤0.70 cm^2^/m^2^) and severe PPM (≤0.60 cm^2^/m^2^) [10]. Paravalvular AR can be graded as none/trace, mild, moderate or severe. Importantly, non-SVD might accelerate the development of SVD.

Bioprosthetic valve thrombosis is a possible complication of bioprosthetic valve replacement and can further lead to BVD. Valve thrombosis can be classified as either clinical or subclinical. Clinical valve thrombosis is defined as symptomatic obstructive valve thrombosis resulting in increased valvular gradients and reduced EOA in echocardiography. The subclinical form of valve thrombosis is characterized by hypoattenuated leaflet thickening (HALT) and reduced leaflet motion (RLM) seen on high-resolution four-dimensional (4D) cardiac computed tomography (CT). HALT can be defined as visually identified increased leaflet thickness with typical meniscal appearance. The main significance of HALT/RLM is that it may represent a potential mechanism of BVD and may provide a possible target for affected valve durability. Subclinical thrombosis is asymptomatic and is usually seen with normal transvalvular gradients at echocardiography. Subclinical thrombosis seems to be common in bioprosthetic valves. The PARTNER 3 CT substudy estimated an overall incidence of HALT of 10% at 30 days and 24% at 1 year follow-up. Even though these findings led to a minimal increase in aortic valve gradients, the clinical consequences need further investigation [11].

Another potentially reversible cause of BVD is infective endocarditis (IE). Endocarditis can be diagnosed according to the modified Duke Criteria [12]. The treatment of IE is based on specific antibiotic therapy. In case of unsuccessful antibiotic therapy, large vegetations, severe valve deterioration, abscess formation or emboli, surgical treatment should be considered [13].

### 2.2. VIVID Definition

One year after the introduction of the EAPCI definitions, a similar definition of SVD was proposed by the VIVID group [7]. SVD is interpreted as a gradual process with different stages. Stage 0 is seen as no significant change from post-implantation state. Morphological leaflet abnormalities such as thickening, fluttering or asymmetrical opening and closure without haemodynamic changes are seen as Stage 1. Stage 2 SVD after exclusion of thrombosis refers to abnormalities with moderate hemodynamic dysfunction and should be divided into Stage 2S (increase in transvalvular gradient ≥ 10 mmHg with a concomitant decrease in valve area in case of stenosis), Stage 2R (in case of regurgitation) and Stage 2RS (in case of mixed moderate stenosis/regurgitation). Stage 3 refers to the most severe stage of SVD and is not separated into stenosis or regurgitation [7].

### 2.3. VARC-3 Definition

Just recently, the VARC-3 committee introduced their updated definition of BVD and BVF. Similar to the EAPCI consensus paper, BVD is described as SVD, NSVD, thrombosis or endocarditis.

For hemodynamic alteration, VARC-3 introduced the term hemodynamic valve deterioration (HVD) that can be utilized for all subclasses of BVD, which is new, compared to previous definitions. There is a detailed subclassification for SVD. Stage 1 refers to morphological valve deterioration without hemodynamic changes. Stage 2 (moderate HVD) is classified as hemodynamic changes together with an increase in mean transvalvular gradient ≥ 10 mmHg, resulting in mean gradient ≥ 20 mmHg with concomitant decrease in aortic valve area (AVA) ≥ 0.3 cm^2^ or ≥25%, and/or decrease in Doppler velocity index (DVI) ≥ 0.1 or ≥20% compared to baseline (up to 3 months post-procedure) or new/increase in transvalvular AR ≥ 1, resulting in moderate transvalvular AR. Stage 3 is defined as severe HVD and refers to an increase in mean transvalvular gradient ≥ 20 mmHg, resulting in mean gradient ≥ 30 mmHg with concomitant decrease in AVA ≥ 0.6 cm^2^ or ≥50%, and/or decrease in DVI ≥ 0.2 or ≥40% compared to baseline or new/increase in transvalvular AR ≥ 2, resulting in severe AR.

NSVD is described as any extrinsic abnormality resulting in valve dysfunction, such as residual para-prosthetic AR or PPM. Thrombosis is defined as subclinical or clinical valve thrombosis. Subclinical thrombosis is characterized by imaging findings of HALT or RLM with absent or mild hemodynamic changes and no symptoms or sequelae. Thrombosis is defined as clinically significant if there are clinical sequelae of thromboembolic events or of worsening AR/AS together with hemodynamic valve deterioration (HVD) stage 2–3 or confirmatory imaging of HALT/RLM. In the absence of clinical sequelae, there should be both HVD Stage 3 and confirmatory imaging of HALT/RLM. Endocarditis is described similar to the EAPCI definition.

BVF is divided into three stages. Stage 1 describes any valve dysfunction associated with clinically expressive criteria or irreversible stage 3 HVD, including NSVD, thrombosis or endocarditis as potential causes. Stage 2 is described as the need for re-operation or re-intervention and stage 3 remains related to valve-related death [6].

**Figure 1:**
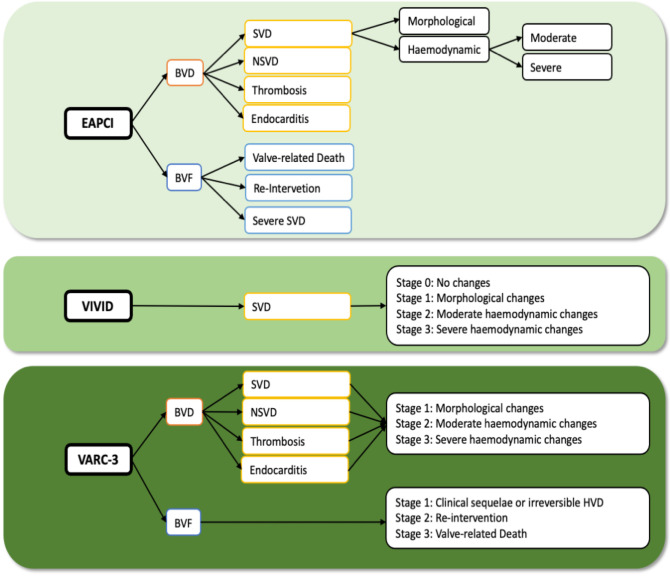
Simplified illustration of current definitions on durability of transcatheter heart valves. EAPCI, European Association of Percutaneous Cardiovascular Interventions; BVD, bioprosthetic valve dysfunction; BVF, bioprosthetic valve failure; SVD, structural valve deterioration; NSVD, non-structural valve deterioration; VIVID, Valve in Valve International; VARC-3, Valve Academic Research Consortium 3; HVD, hemodynamic valve deterioration.

A simplified illustration of the current definitions is shown in Figure 1.

## 3. Long-Term Durability of Transcatheter Heart Valves

Long-term durability seems to be the main limitation of bioprosthetic valves. In comparison to SAVR, reports on long-term durability for TAVR are still scarce. This is mainly due to the older age of the TAVR population, which is classically characterized by a high-risk profile, multiple comorbidities and a limited life expectancy. Theoretically, durability of THV can differ from that of surgical valves due to the possible trauma during initial valve preparation and compression, balloon dilatation or asymmetrical frame expansion.

Numerous studies have reported an acceptable hemodynamic function and low rates of SVD up to 5 years [14,15], but reports on long-term outcomes beyond 5 years are still limited. An important challenge when reporting data on long-term durability deals with the definition of specific criteria. After the introduction of the EAPCI definitions in 2017 [8], several studies published their data on durability of THV with a follow-up of more than 5 years. An overview on the available data is shown in Table 1.

### 3.1. 5-Year Outcomes after TAVR

Several studies have reported on 5-year durability outcomes after TAVR. Pibarot et al. [16] recently compared the 5-year incidence of SVD in the PARTNER 2A trial and the observational SAPIEN 3 trial. SVD and BVF were defined according to the VARC-3 definitions without using cumulative incidence function. In the first comparison (Sapien XT vs. SAVR in the randomized cohort), the adjusted incidence rate (per 100 patient-years) of SVD and SVD-related BVF was significantly higher in the TAVR group compared to the matched SAVR group (1.61% and 0.58%, respectively, *p* < 0.01). In a second comparison, the exposure adjusted incidence rate of SVD and SVD-related BVF in the observational Sapien 3 cohort was 0.68% and 0.29%, respectively, and showed no difference compared to a matched SAVR cohort. Comparing the Sapien 3 cohort with the Sapien XT cohort, there were significantly lower exposure adjusted incidence rates of SVD and SVD-related BVF in the Sapien 3 cohort.

Contrary results were found in the 5-year follow-up of the CoreValve U.S. Pivotal High Risk Trial, reported by Gleason et al. [17]. Moderate SVD (defined according to EAPCI) was significantly more common in SAVR compared to TAVR (26.6% vs. 9.5% *p* < 0.001), using actuarial analysis. There was no significant difference in the occurrence of severe SVD between both groups. Specific actual analysis on SVD was not performed. In both studies, there were no data on endocarditis and NSVD reported.

Abdel-Wahab et al. [15] recently reported the 5-year follow-up data from the CHOICE randomized clinical trial and found a significantly higher cumulative incidence of moderate/severe SVD (defined according to EAPCI) in balloon-expandable (BE) valves (6.6%) compared to self-expanding (SE) valves (0%). The incidence of BVD was similar between both groups (BE valves 22.5% vs. SE valves 20%, *p* = 0.91). Similarly, for NSVD, there was no statistically significant difference between both groups (BE 17.8% vs. SE 26.7%, *p* = 0.20). The cumulative incidence of clinical valve thrombosis was numerically higher with BE valves (7 BE valves (7.3%) vs. 1 SE valve (0.8%)). Endocarditis occurred in 1.6% of BE valves and in 3.4% of SE valves. The rate of BVF was low and not significantly different between both groups (4.1% vs. 3.4%, *p* = 0.63%).

Reports from national registries confirm low rates of long-term valve dysfunction after TAVR. Didier et al. [14] reported the 5-year clinical outcomes of the FRANCE-2 registry. Defining SVD according to the EAPCI consensus statement, they found a 13.3% cumulative incidence of moderate SVD and a 2.5% cumulative incidence of severe SVD. The rate of endocarditis was low and ≤0.3% after the first year. Data on BVF, NSVD and thrombosis were not reported.

### 3.2. 5–10-Year Outcomes after TAVR

A total of four single-center studies and one multi-center study showed stable trans-prosthetic gradients and low rates of SVD after 8-year follow-up.

Eltchaninoff et al. [18] found a cumulative incidence of SVD and BVF of 3.2% and 0.58%, respectively, in patients with BE valves after 8 years. In a study by Barbanti et al. [19], the cumulative incidence function of moderate and severe SVD (according to the EAPCI definition) was 5.87% and 2.3%, respectively. BVF occurred in 4.51%. The survival rates free from BVF and severe SVD at 8 years were 95.4% and 97.5%.

Aldalati et al. [20] showed results of a retrospective follow-up analysis of up to 8 years using different definitions. SVD was defined according to VARC-2 [10], VIVID [7] and the EAPCI [8]; definitions and data were reported separately for each definition. The EAPCI definition of SVD was modified in terms of including new or increasing paravalvular and intraprosthetic regurgitation as part of SVD. SVD rates were compared using Kaplan-Meier estimates. According to the VARC-2 and VIVID definition, the rate of SVD was similar between both groups (TAVR 28% vs. SAVR 31%, *p* = 0.593; TAVR 11.5% vs. SAVR 19%, *p* = 0.022). When applying the EAPCI definition, moderate SVD was significantly more common among the SAVR group (TAVR 11.5% vs. 20.7% *p* = 0.007). Severe SVD was similar between both groups (TAVR 2.2% vs. SAVR 1.7%, *p* > 0.099). Patients with endocarditis were excluded. There were no reports on NSVD and thrombosis cases.

Blackman et al. [21] reported a <0.5% incidence of severe SVD at a median follow-up of up to 10 years from the UK TAVI Registry. The cumulative incidence of moderate SVD was 8.7%. One patient (0.4%) developed a severe intraprosthetic AR at 5 years and 4 months after procedure who had only mild PVL at baseline. There was no change in the incidence of moderate AR during follow-up. Data on NSVD, thrombosis and endocarditis were not reported. 

Similar results were shown by Jørgesen et al. from the NOTION trial, which is the first study providing long-term data on patients with low surgical risk [22]. The 8-year analysis, where 145 TAVR patients and 135 SAVR patients were compared, showed that the rate of SVD was numerically higher for SAVR than for TAVR (8.8% vs. 15.7%, *p* = 0.068) without significant difference. BVD occurred in 62.0% of TAVR patients and 70.5% of SAVR patients with no significant difference. NSVD occurred mainly due to PPM and was significantly higher in patients treated with SAVR (TAVR 43.9% vs. SAVR 60.7%, *p* = 0.0049). The rate of endocarditis was similar between both groups (TAVR 7.2% vs. SAVR 7.4%, *p* = 0.95). There were no cases of thrombosis observed. The incidence of BVF at 5 years was low and did not differ significantly between both groups (TAVR 8.7% vs. SAVR 10.5%, *p* = 0.61).

BVF was defined according to the EAPCI consensus statement. Jorgesen et al. presented a modification of the consensus statement for SVD and NSVD since they tried to account for a high proportion of SAVR patients treated with small valves who show elevated mean gradient early after implantation. Therefore, the authors combined having a mean gradient ≥ 20 mmHg and an increase in the mean gradient ≥ 10 mmHg and extended the time for baseline measurement to 3 months.

### 3.3. ≥10-Year Outcomes after TAVR

Very recently, Sathananthan et al. [23] showed the very first results of a 10-year follow-up of 235 patients receiving THV. The cumulative incidence of subjects having SVD and BVF was 6.5% and 2.5% at 10 years. Nine patients had moderate SVD and six patients had severe SVD, where two had to undergo reinterventions. There was no difference between transvalvular gradient at discharge and at 10-year follow-up. SVD was defined as per EAPCI consensus statement. BVF was modified from the consensus statement and was defined as the rate of valve reintervention and severe hemodynamic SVD. Results were reported as a cumulative incidence to account for the competing risk of death. Data on thrombosis, NSVD or endocarditis were not reported.

**Table 1:** Summary of studies reporting data on TAVR long-term durability.

Author	FU (Years)	Sample (*n*)	Key Findings
Pibarot, P. [16] **	5 y	Sapien XT TAVR (*n* = 774) Sapien 3 TAVR (*n* = 891) SAVR (*n* = 664)	Sapien XT cohort vs. SAVR SVD (1.61% vs. 0.63%, *p* < 0.01) SVD related BVF (0.58% vs. 0.12%, *p* < 0.01) Sapien 3 vs. Sapien XT SVD (0.63% vs. 1.76%, *p* = 0.0001) SVD related BVF (0.21% vs. 0.65%, *p* = 0.03) Sapien 3 vs. SAVR: SVD (0.68% vs. 0.60%, *p* = 0.71) SVD related BVF (0.29% vs. 0.14%, *p* = 0.25)
Jørgesen, T.H. [22] *,**	8 y	TAVR (145) SAVR (135)	BVD (TAVR 62.0% vs. SAVR 70.5%, *p* = 0.064) SVD (TAVR 8.8% vs. SAVR 15.7%, *p* = 0.068) NSVD (TAVR 43.9% vs. SAVR 60.7%, *p* = 0.0049) Thrombosis (0%) Endocarditis (TAVR 7.2% vs. SAVR 7.4%, *p* = 0.95) BVF (TAVR 8.7% vs. SAVR 10.5%, *p* = 0.61)
Aldalati, O. [20] *,**	6.5 y	269 TAVR 174 SAVR	Moderate SVD (TAVR 11.5% vs. SAVR 20.7%, *p* = 0.007)
Gleason, T.G. [17] *	5 y	391 (TAVR) 359 (SAVR)	Moderate SVD (TAVR 9.5% vs. SAVR 26.6%, *p* < 0.001) Severe SVD (TAVR 0.8% vs. SAVR 1.7%, *p* = 0.32)
Testa, L. [24] **	8 y	990 TAVR	Moderate SVD (3.0%) Severe SVD (1.6%) Late BVF (2.5%)
Abdel-Wahab, M. [15] *	5 y	BE TAVR (121) SE TAVR (120)	BVD (BE 22.5% vs. SE 20%, *p* = 0.91) SVD (BE 6.6% vs. SE 0%, *p* = 0.018) NSVD (BE 17.8% vs. SE 26.7%, *p* = 0.20) Thrombosis (BE 7.3% vs. SE 0.8%, *p* = 0.06) Endocarditis (BE 1.6% vs. SE 3.4%, *p* = 0.39) BVF (BE 4.1% vs. SE 3.4%, *p* = 0.63)
Sathananthan, J. [23] *,**	10 y	235 TAVR	SVD (6.5%) BVF (2.5%)
Murray, M.I. [25] *	7 y	103 TAVR	BVF (3.8%) Severe SVD (1.3%) Moderate SVD (8.9%) Thrombosis (1.3%) Endocarditis (1.3%)
Durand, E. [26] *	7 y	1403 TAVR	Moderate SVD (7.0%) Severe SVD (4.2%) BVF (1.9%)
Orvin, K. [27] *,**	5 y	450 TAVR	SVD (12.3%) BVF (0.6%) annualized incidence BVD (1.8%) annualized incidence
Panico, R.A. [28] *	7 y	278 TAVR	SVD 3.6% BVF 2.5% Thrombosis (0%)
Blackman, D.J. [21] *,**	5.8 y	241 TAVR	Severe SVD (<0.5%) Moderate SVD (8.7%)
Eltchaninoff, H. [18] *	8 y	378 TAVR	SVD (3.2%) Late BVF (0.58%)
Deutsch, M.-A. [29] *	7 y	300 TAVR	SVD (14.9%) BVF (*n* = 10)
Barbanti, M. [19] *	8 y	286 TAVR	Severe SVD (2.30%) Moderate SVD (5.87%) BVF (4.51%) Thrombosis (0%)
Didier, R. [14] *	5 y	4187 TAVR	Moderate SVD (13.3%) Severe SVD (2.5%)
Holy, E.W. [30] *	8 y	152 TAVR	BVF (4.5%) Severe/moderate SVD (0%)

Y, years; SE, self-expandable valve; BE, balloon-expandable valve; TAVR, transcatheter aortic valve replacement; SAVR, surgical valve replacement; FU, follow-up; SVD, structural valve deterioration; NSVD, non-structural valve deterioration; BVD, bioprosthetic valve dysfunction; BVF, bioprosthetic valve failure; PVL, paravalvular leakage; PVR, prosthetic valve regurgitation. * EAPCI/ESC/EACTS definition on valve durability, ** modified definition.

## 4. Misconceptions in Reporting Long-Term Results on TAVR Durability

**Figure 2:**
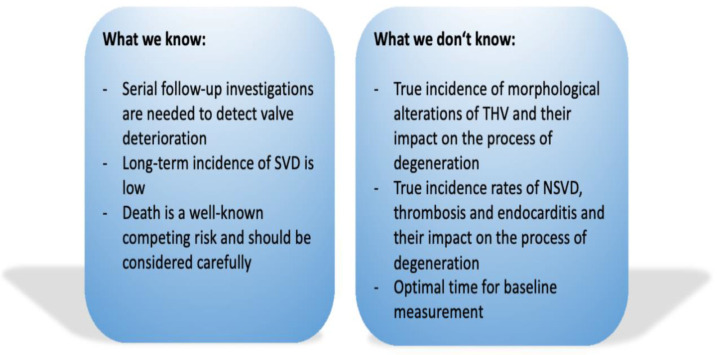
Simplified illustration of current knowledge and future aspects in durability of transcatheter heart valves. SVD, structural valve deterioration; THV, transcatheter heart valve; NSVD, non-structural valve deterioration.

In recent years, many studies were published reporting long-term data of TAVR patients. Overall, the results seem to be excellent for TAVR procedures with low rates of SVD and non-inferiority compared to SAVR. However, even though there were improvements in the past, there are still some issues and misconceptions concerning long-term durability of TAVR (Figure 2).

### 4.1. Definitions

The EAPCI proposed a standardized definition for BVD to create uniformity in interstudy comparisons. This guideline included transcatheter and surgically implanted bioprosthetic valves. However, it seems that this standardized definition is not accurately used by many authors. One year after the presentation of the EAPCI definition on bioprosthetic valve durability, the VIVID group proposed a more staged definition of SVD. Just recently, the VARC-3 definition stated an updated definition of BVD and BVF, including DVI and EOA as additional parameters and introducing HVD as a new parameter, representing hemodynamic changes in SVD, NSVD, thrombosis and endocarditis. Still, there is no consensus on an updated version, harmonizing the different suggestions.

#### 4.1.1. Structural Valve Deterioration

Even though the definition on SVD seems to be a widely accepted part of the EAPCI definition of BVD, there are still some noticeable deviations. Some authors are using invalid definitions of SVD, since they extend the time of baseline measurement [22] and combine different entities to define SVD [22,24]. None of the studies commented on morphological changes although these are fundamental in the recognition of SVD [7]. Furthermore, some did not differentiate between severe and moderate SVD [22], which makes it difficult defining BVF later on.

Different authors criticized the EAPCI definition concerning commonly higher gradients in surgically implanted biological valves. Higher gradients seen in certain SAVR patients may not be indicative for true SVD since it is mainly caused by factors such as size of the valve or PPM. A post-procedural gradient of 19 mmHg at baseline that is thereafter measured as 20 mmHg would meet the criteria of moderate SVD. Modified criteria in means of combining elevated gradient with an increase in gradient are suggested to be a better definition [17,27]. Recently, Hahn et al. stated that an increase in mean gradient > 10 mmHg is not enough to define SVD, which should be accompanied by concomitant decrease in EOA >25% and/or DVI >20% [31]. Since these factors are included in the updated VARC-3 definition, future results remain to be seen.

#### 4.1.2. NSVD, Thrombosis and Endocarditis

Other causes potentially leading to BVD are NSVD, thrombosis and endocarditis. Long-term data on NSVD, thrombosis and endocarditis are still limited and not well reported in the present studies. Most of the studies reported data on SVD but did not exclude thrombosis as a potential cause of increased transvalvular gradient. Since valve thrombosis could be associated with accelerated SVD, there should be further investigations on the diagnosis, the cause and the treatment of subclinical and clinical valve thrombosis. Abnormal blood flow within the neo-sinus has been implicated as a potential mechanism [32].

Furthermore, it seems to be important to keep in mind that SVD and NSVD as well as endocarditis and thrombosis are not mutually exclusive processes. In particular, all processes can exist side by side and can accelerate the development of SVD due to altered valve hemodynamics and mechanical stress [33,34,35]. Therefore, clear differentiation and indication for further investigations are needed.

Since it is well known that it may be difficult to differentiate between thrombosis and SVD in case of elevated transvalvular gradients, the incidence of SVD might be overestimated. Transthoracic echocardiography (TTE) seems to present certain limitations and is not always able to discriminate between central and paravalvular regurgitation or between SVD and valve thrombosis. These cases may require further investigations using CT to overcome the limitations of TTE. Detailed statements on clinical and subclinical thrombosis and the use of CT were shown in the updated VARC-3 definition, whereby future studies applying these new definitions are expected.

From all the screened studies, only two studies reported data on NSVD [15,22]. According to the EAPCI consensus statement, NSVD may occur early after TAVR as a result of technical issues and may not increase over time. Even though factors such as PPM and PVL seem to be clearly defined elsewhere [36], there is a lack of a specific time span of NSVD to occur. Furthermore, intra-prosthetic or PVL were classified as NSVD whereas new or increased intraprosthetic regurgitation also qualified as SVD. In the updated VARC-3 definition, NSVD is clearly classified as PPM, PVL or other extrinsic factors [6]. In future studies, there should be a clear discrimination between those parameters.

### 4.2. Death as a Competing Risk

Since the studied cohorts mainly belong to an older population within a high-risk patient group, death seems to be a competitive risk factor against the risk of a valve to fail over time. In this context, conventional Kaplan–Meier analysis (a type of actuarial analysis) may lead to incorrect estimates. The EAPCI consensus statement and the updated VARC-3 definition recommended using cumulative incidence function as an actual method to report the correct probability. This method provides lower estimates than actuarial analysis and might have greater clinical utility in the context of TAVR durability studies [6,7].

Not all the studies published from 2017 onwards used cumulative incidence functions for their estimates. In six out of the seventeen reviewed articles, cumulative incidence function was not used to account for death as a competing risk in their estimates. This raises major concerns about the reported data and makes a reliable interstudy comparison almost impossible.

### 4.3. TAVR as a New and Quickly Developing Technique

The interpretation of the present TAVR literature still warrants some discussion. There are several factors that prevent robust evaluation of TAVR durability. First, the major factor is the older age of the TAVR population with many comorbidities resulting in a limited life expectancy, and therefore, a paucity of patients is available for long-term evaluation. Second, there have been many improvements and a rapid turnover from one device generation to the next during the time from the first implantation in 2002. Current data on long-term outcomes refer to first-generation valves, which were implanted with low operator experience with high rate of valve malpositioning and sizing problems. Preoperative CT diagnostics and new types of valves improved that problem. The past years have brought about major procedural improvements owing to advances in imaging and patient selection, operator experience and technological improvements, including advances in stent frame technology and anti-calcific and anti-immunogenicity treatments, which could improve long-term durability for more recently implanted valves. Therefore, analyzing data using older techniques and technologies could be misleading and underestimate the long-term durability of transcatheter heart valves.

Authors should discuss the results and how they can be interpreted from the perspective of previous studies and of the working hypotheses. The findings and their implications should be discussed in as broad a context as possible.

## 5. Conclusions

Since TAVR is further expanding to younger patients with longer life expectancy, it is essential to report further data on long-term durability. The first studies reporting SVD up to 10 years after implantation provide encouraging results. Further evaluation of long-term valve function using standardized definitions remains essential.

Existing data show the complexity in reporting accurate information on long-term durability of THV. Despite the multidisciplinary approach of the 2017 EAPCI recommendations and the advances in defining bioprosthetic valve durability that came with it, there are still limitations regarding the general applicability. Lately, the VARC-3 writing committee published a detailed standardized definition addressing current issues to create uniformity when reporting and comparing interstudy results on TAVR durability [6].

In future studies, we suggest more detailed descriptions of SVD, NSVD, thrombosis and endocarditis. First, there should be more emphasis on morphological SVD. For hemodynamic deterioration in case of AS, definitions should include a combination of total transvalvular gradient and an increase in transvalvular gradient and should further include EAO and DVI as additional parameters to create harmonization. For identifying thrombosis and differentiating thrombosis from SVD in case of elevated gradients seen on echocardiography, CT should be performed. NSVD should be clearly defined as increased gradients early after implantation of the valve. The time span should be limited to 30 days after implantation, since this time span was defined as baseline measurement by the EAPCI recommendations [8]. Furthermore, competing risks should be routinely taken into account in all statistical estimates.

To report accurate data on TAVR durability, it remains vital that future registries are conducted according to high standards with validated standardized echocardiographic core labs, consecutive recruitment with serial measurements, accurate prospective long-term follow-up with documentation of all relevant adverse events, along with a statement of completeness of follow-up.

The integration of these approaches may contribute to significant improvements and could partly overcome the challenges in scientific and clinical research on transcatheter valve function to provide more reliable data on long-term durability.
